# The Many and Varied Roles of Tetraspanins in Immune Cell Recruitment and Migration

**DOI:** 10.3389/fimmu.2018.01644

**Published:** 2018-07-18

**Authors:** Louisa Yeung, Michael J. Hickey, Mark D. Wright

**Affiliations:** ^1^Centre for Inflammatory Diseases, Department of Medicine, Monash Medical Centre, Monash University, Clayton, VIC, Australia; ^2^Department of Immunology, Monash University, Prahran, VIC, Australia

**Keywords:** tetraspanin, leukocyte migration, adhesion molecules, inflammation, integrins

## Abstract

Immune cell recruitment and migration is central to the normal functioning of the immune system in health and disease. Numerous adhesion molecules on immune cells and the parenchymal cells they interact with are well recognized for their roles in facilitating the movements of immune cells throughout the body. A growing body of evidence now indicates that tetraspanins, proteins known for their capacity to organize partner molecules within the cell membrane, also have significant impacts on the ability of immune cells to migrate around the body. In this review, we examine the tetraspanins expressed by immune cells and endothelial cells that influence leukocyte recruitment and motility and describe their impacts on the function of adhesion molecules and other partner molecules that modulate the movements of leukocytes. In particular, we examine the functional roles of CD9, CD37, CD63, CD81, CD82, and CD151. This reveals the diversity of the functions of the tetraspanin family in this setting, both in the nature of adhesive and migratory interactions that they regulate, and the positive or inhibitory effects mediated by the individual tetraspanin proteins.

## Introduction

The ability of leukocytes to migrate from the circulation to sites of inflammation is essential for effective host defense. To undertake this journey, leukocytes undergo a series of interactions in the bloodstream with endothelial cells lining the vasculature (1, 2). The critical roles of cell surface-expressed adhesion molecules on leukocytes and vascular endothelial cells in mediating these interactions are well established. Less appreciated is the emerging evidence indicating important contributions for members of the tetraspanin family of cell membrane proteins in this process. Tetraspanins work differently to classical adhesion molecules; they do not have ligands on other cells, but regulate the actions of target molecules *in cis*, i.e., expressed in the same cell. In this review, we will analyze the developing knowledge on the role of tetraspanins in controlling the movements of immune cells.

## Leukocyte Recruitment is a Sequential, Multistep Process

**Figure 1:**
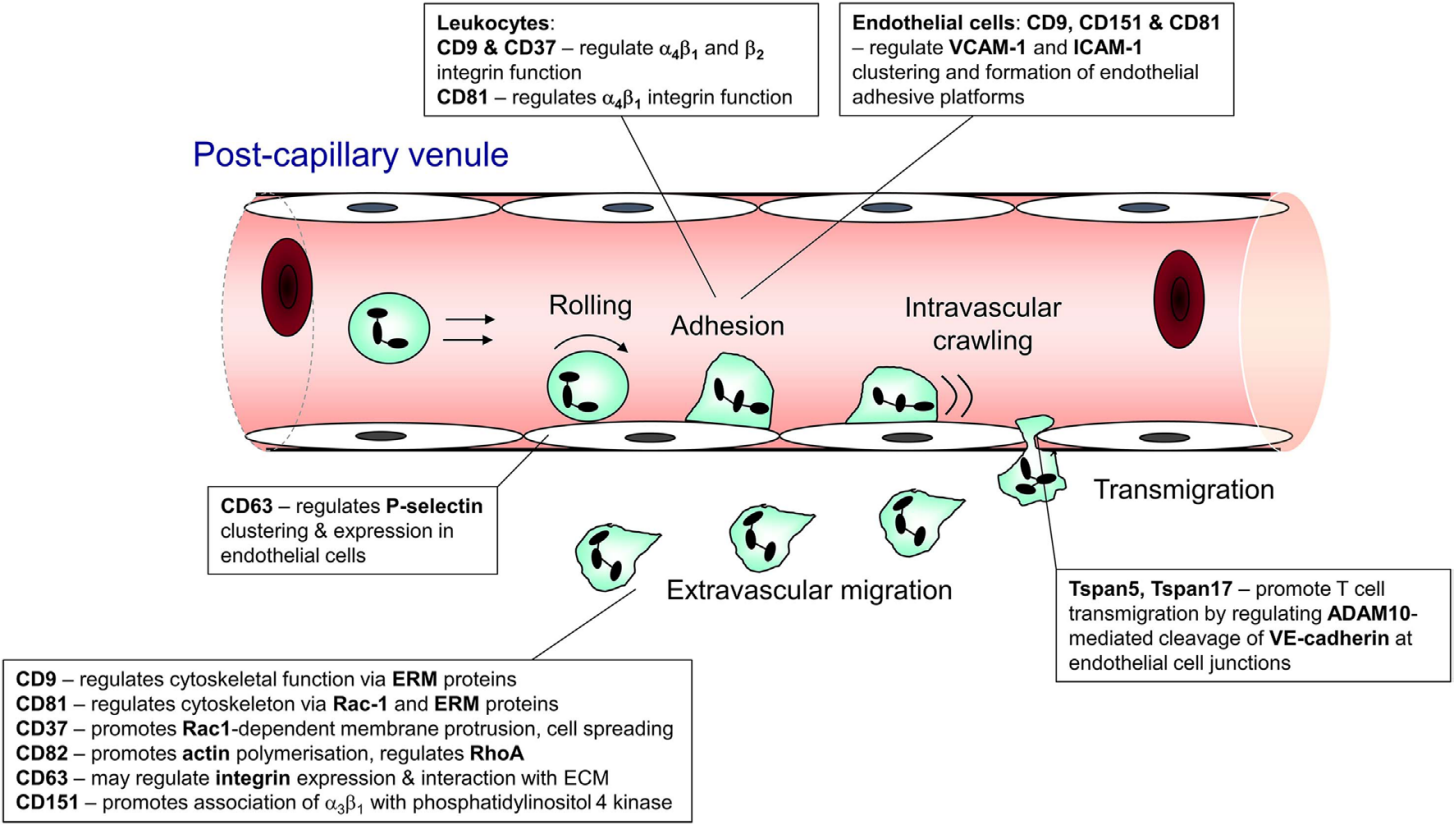
Steps in leukocyte trafficking influenced by tetraspanins. Image shows the sequence of interactions undergone by leukocytes during their recruitment from the bloodstream and after they exit the vasculature, with the tetraspanins that influence these interactions shown adjacent to the interaction. This information is taken from the following publications: CD63 (4, 5); CD9 (6, 7); CD37 (8, 9); CD81 (10, 11); Tspan 5 and 17 (12); CD82 (9); and CD151 (13, 14).

Recruitment of leukocytes from the circulation is essential both to homeostatic immune surveillance and the response to infection and injury. In innate inflammation, neutrophils and monocytes undergo rapid recruitment to the affected site to mediate the appropriate response (1). Similarly, in adaptive immunity, the recirculation and trafficking of B and T lymphocytes is crucial to ongoing surveillance against potential invading pathogens (2). In both cases, leukocytes leave the bloodstream *via* a sequence of steps, collectively known as the leukocyte recruitment cascade. This involves interactions mediated by an array of adhesion molecules that function cooperatively to arrest the cell on the endothelial surface and facilitate its transmigration into the surrounding tissue (3). The main sequential steps in leukocyte recruitment are rolling, adhesion, crawling, and transmigration (1, 3), and tetraspanin family members have been shown to contribute to each of these steps (Figure 1).

## Selectins Mediate Early Interactions During Leukocyte Recruitment

The initial interactions between leukocytes and the activated vascular endothelium are mediated by the selectins. The selectin family consists of three members; L-selectin, which is constitutively expressed on leukocytes (15), and E- and P-selectin, found on activated endothelial cells (16–18). The selectins show overlapping properties and are able to interact with ligands such as P-selectin glycoprotein ligand 1 *via* recognition of the crucial SLe^x^ carbohydrate motif (19–21). The rapid on–off interactions mediated by selectins and their ligands allow for the initial capture of rapidly moving leukocytes in the bloodstream and their subsequent rolling along the vessel wall (22–26).

## Integrins Mediate Arrest of Leukocytes on the Endothelium

Leukocyte integrins are the main adhesion molecules responsible for mediating leukocyte firm adhesion to the endothelium. G protein-coupled chemoattractant receptors expressed on the surface of rolling leukocytes are able to detect and respond to chemoattractants present within the microvasculature (27, 28). These signals rapidly (sub-second) induce integrins to undergo a conformational change from a low affinity to high affinity form, leading to integrin-dependent arrest of the leukocyte (27, 29, 30). The key integrins on circulating leukocytes are the β_2_ integrins LFA-1 (α_L_β_2_) and Mac-1 (α_M_β_2_), which interact with their ligands on endothelial cells including ICAM-1 and ICAM-2, and the α_4_ integrins VLA-4 (α_4_β_1_) and α_4_β_7_ which interact with VCAM-1 and MAdCAM-1, respectively (3, 31). After arrest, integrin-mediated outside-in signaling promotes the strengthening of adhesion to the endothelium (3, 32).

## Intravascular Crawling and Transmigration

Integrins also contribute to processes downstream of leukocyte adhesion, particularly intraluminal crawling and transmigration. Upon integrin binding, signal transduction results in the alteration of the internal dynamics of the cell; cytoskeletal changes allow for pseudopodia formation and intraluminal crawling along the endothelium. Crawling allows leukocytes to scan the endothelium for suitable locations for transmigration (33). Transmigration occurs predominantly at inter-endothelial cell junctions (paracellular transmigration), where leukocytes initiate transmigration *via* extension of uropods into the junction before migrating through. While paracellular migration is the predominant mode of transendothelial migration, under some circumstances leukocytes cross the endothelial barrier by migrating directly through endothelial cells, in what is termed transcellular migration (34, 35). Various adhesion molecules have roles in transmigration, including PECAM-1, CD99, JAM-A, β_2_ and β_1_ integrins, and L-selectin (36–39). Leukocytes then migrate through the interstitium by following a chemotactic gradient to the source of inflammation, a process involving further interactions of leukocyte integrins with extracellular matrix (ECM) ligands (3).

## Immune Cell Migration and the Cytoskeleton

Immune cell motility and directional migration requires the formation of lamellipodia at the leading edge with adhesion to ECM matrix proteins, while simultaneously there is a requirement for detachment at the trailing edge (40). These tightly regulated events require coordinated assembly and disassembly of actin and myosin filaments, processes heavily influenced by members of the Rho family of GTPases. Here, Rac1 regulates actin polymerization at the lamellipodia, while RhoA influences the contraction of actin at the rear of the cell, allowing for forward movement. Meanwhile, evidence indicates Cdc42 is involved in controlling the direction of migration (40).

Dendritic cell (DC) migration is essential for the initiation of the adaptive immune response and exemplifies the importance of cytoskeletal rearrangement in immune cell migration. Here, migration is driven by chemotactic gradients that guide DCs in the interstitium to the lymphatic microvasculature en route to local draining lymph nodes (41). The role of adhesion molecules in DC migration is less clear, with evidence supporting both adhesion-dependent and -independent modes of migration (42). For the latter mode, it is apparent that actin polymerization and cytoskeletal rearrangement are of critical importance (43).

## Tetraspanins: Organizers of the Surface Membrane

Successful interactions between a receptor–ligand pair result in the generation of intracellular signals that alter the environmental dynamics of the cell. However, for receptors to productively interact with their ligands and efficiently transduce signals, they must be organized at the cell surface. Tetraspanins are a family of 33 membrane proteins (in humans) which are central to this membrane organization (44). Tetraspanins have the ability to interact and cluster with an array of tetraspanin and non-tetraspanin partners within the cell membrane, forming organized networks of signal transducing complexes termed tetraspanin-enriched microdomains (TEMs) (45–48).

Tetraspanins are distinguished from other four transmembrane proteins by the presence of key conserved amino acid residues located in the transmembrane regions, as well as in the large extracellular loop (LEL) (48). At least in the recently solved X-ray structure of the tetraspanin CD81, the four transmembrane domains form alpha helices that create an intramembrane cholesterol-binding pocket (49). Historically, the LEL of tetraspanins has been the predominant structure studied as it contains the sites responsible for generating protein–protein interactions (45). In addition, much attention has focused on the cytoplasmic domains which can interact with signaling molecules and contain conserved membrane-proximal cysteine residues that are palmitoylation sites (48) which aid in the stabilization of tetraspanin–tetraspanin interactions (50), and contribute to the formation of the TEMs (48).

## The Diversity of Tetraspanin Interactions

Although tetraspanins lack conventional ligands, they interact with a diverse assortment of molecules within the TEM (51). Recent super-resolution microscopy studies indicate a considerable heterogeneity among TEMs, in that tetraspanins such as CD53 form nanoclusters in the plasma membrane, and are more likely to be directly associated with non-tetraspanin partners than with other tetraspanin family members (52). This diversity of molecular interactions and heterogeneity of microdomains explain the pleiotropic functions a single tetraspanin may play. CD81 is an excellent example: in macrophages CD81 suppresses cell growth (53), while in B cells, CD81 regulates CD19 expression, lowering the threshold for activation, and promotes adhesiveness of the α_4_β_1_ integrin (10, 54). In T cells, CD81 interacts with CD3ζ of the TCR, regulating T cell activation in response to antigen recognition (55) as well as controlling sustained T cell activation following antigen presentation through interactions with both CD3ζ and ICAM-1 (56).

## Role of Tetraspanins in Immune Cell Migration and Recruitment

**Figure 2:**
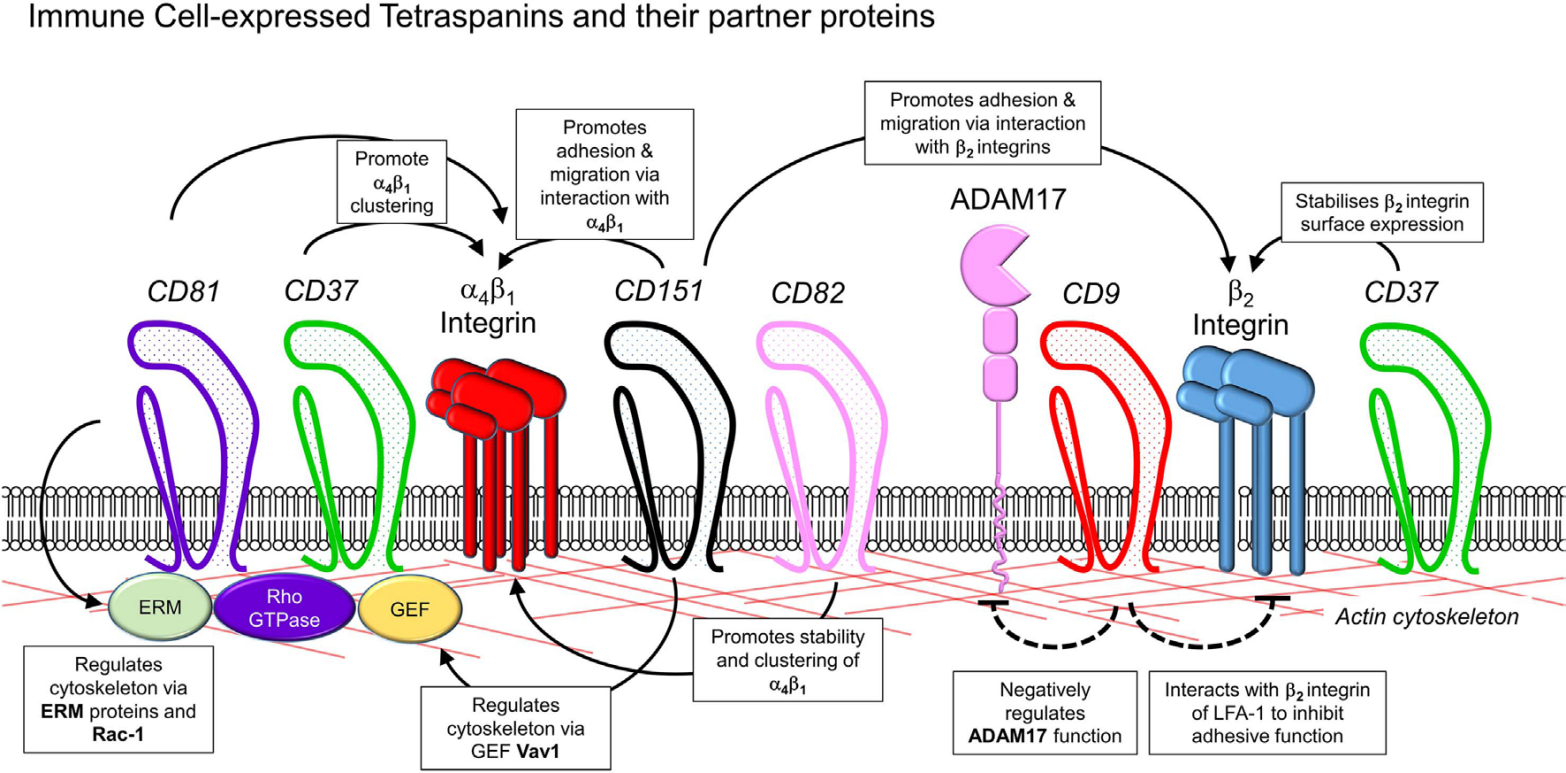
Interactions of leukocyte-expressed tetraspanins and co-expressed molecules involved in leukocyte trafficking. Interactions of tetraspanins expressed in immune cells can occur with other tetraspanins, along with members of several other families of molecules involved in control of adhesion and cytoskeletal function. These include β_1_ and β_2_ integrins, metalloproteases such as ADAM17, adhesion molecules of the immunoglobulin superfamily such as ICAM-1, the actin cytoskeleton, and intracellular signaling molecules such as guanosine exchange factors (Vav1, SLP76), Rho GTPases, and ezrin/radixin/moesin proteins.

**Figure 3:**
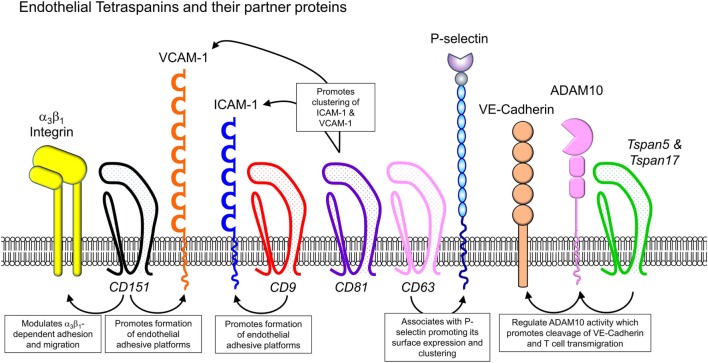
Endothelial cell-expressed tetraspanins and co-expressed proteins relevant to leukocyte trafficking. Endothelial cells play critical roles in directing immune cells from the bloodstream into sites of inflammation or secondary lymphoid organs. Tetraspanins expressed in endothelial cells, including CD9, CD63, CD81, CD82, CD151, and Tspan5 and Tspan17, have been shown to impact on endothelial cell adhesive function, by regulating the function of various adhesion molecules (integrins, ICAM-1, VCAM-1, and P-selectin) and MMPs such as ADAM10.

The role of tetraspanins in cellular migration has been examined in detail in regards to tumor cells. However, a series of more recent studies now implicate an important role of tetraspanins including CD9, CD37, CD63, CD81, CD82, CD151, and Tspan5 and Tspan17 in immune cell migration (Figure 1) (57). Here, there appears to be two mechanisms at play that are not mutually exclusive. First, many cell membrane-expressed adhesion molecules are tetraspanin-partner proteins, and their adhesive function and downstream intracellular signaling are regulated by tetraspanins (Figures 2 and 3). Second, extracellular signals stimulating migration have to be communicated to the cytoskeleton for cytoskeletal reorganization to occur. Here, tetraspanins may play a key role through their communication with Rho GTPases and other cytoskeleton-associated proteins (Figure 2).

The tetraspanins that influence leukocyte migration and their mechanisms of action are summarized in Table 1. This review will discuss how tetraspanins expressed in immune cells influence adhesion molecule function and immune cell recruitment, and also examine the functions of tetraspanins expressed in endothelial cells, which play an essential role in directing leukocytes as they migrate through the body.

**Table 1:** Roles of tetraspanin family members in leukocyte–endothelial cell interactions, recruitment, and migration.

Tetraspanin	Immune cell expression	Impact on recruitment	Reference
CD9	Monocytes T cells Neutrophils Endothelial cells	Promotes cell motility through regulation of adhesion molecules, e.g., LFA-1 Promotes formation of endothelial adhesive platforms (EAPs)	(7, 58–61)
CD37	B cells Dendritic cells (DCs) Neutrophils	Promotes cell adhesion through regulation of integrins, e.g., α_4_β_1_, β_2_. Facilitates chemokine-directed migration Promotes cell spreading through regulation of integrin–cytoskeleton cross-talk, and/or integrin stability	(8, 62, 63)
CD63	Endothelial cells	Promotes leukocyte rolling on human umbilical vein endothelial cells through expression and clustering of P-selectin	(5)
CD81	NK cells B cells T cells Monocytes DCs Endothelial cells	Promotes cell adhesion through regulation of adhesion molecules, e.g., LFA-1, VLA-4 Promotes cell adhesion through regulation of actin-associated proteins, e.g., Rac1, Ezrin Required for DC migration	(10, 11, 64–68)
CD82	DCs T cells	Reduces cell motility through regulation of cytoskeletal proteins, e.g., RhoA Promotes cell adhesion through regulation of adhesion molecules, e.g., LFA-1, α_4_β_1_	(9, 69–72)
CD151	T cells Neutrophils Endothelial cells	Promotes cell migration through regulation of extracellular matrix binding Promotes cell adhesion through regulation of actin remodeling and formation of EAPs	(13, 14, 58, 59, 68)
Tspan5 and Tspan17 (TspanC8 family members)	Endothelial cells	Promote T cell transmigration *via* regulation of endothelial MMP ADAM10	(12)

## Tetraspanins as Regulators of the Leukocyte Integrins

### β_1_ Integrins

The first publication that provided evidence of a role for tetraspanins in immune cell adhesion and migration was the report that the ectopic expression of the tetraspanin CD9 in B cell lines promoted adhesion and haptotactic migration in fibronectin-coated transwells (6). Since then, no less than four tetraspanins have been convincingly reported to regulate α_4_β_1_ integrin function in various immune or hemopoietic cells: CD37, CD81, CD82, and CD151 (6, 73–77).

In B cells, there are similarities in the way CD37 and CD81 regulate this key integrin. Tetraspanin CD37 is expressed on most lymphoid cells and has particularly high expression on B cells. Under shear flow conditions *in vitro*, CD37 was required for optimal B cell rolling and adhesion on fibronectin and VCAM-1. CD37 was found to colocalize with α_4_β_1_ in clusters within the B cell membrane and to be essential in the formation of high avidity α_4_β_1_ clusters upon ligand binding to VCAM-1 and the subsequent transduction of survival signals through the Akt pathway (62). Similarly, the ubiquitously expressed CD81, which has been shown using biochemical approaches to preferentially interact with α_4_ integrins, is also essential for α_4_β_1_ function (69). CD81 strengthened α_4_β_1_-dependent adhesion of B cells and monocytes to VCAM-1 under flow and promoted multivalent integrin interactions (10). Despite these studies, the *in vivo* implications of CD81-mediated regulation of α_4_β_1_ adhesiveness are yet to be determined—neither CD81 nor CD37 has been reported to have a role in B cell migration. By contrast, the many B cell impairments caused by CD81 deficiency have been attributed to impaired CD19 expression (78, 79).

Tetraspanin CD151 is best known for its regulation of the laminin and fibrinogen-binding integrins α_3_β_1_, α_6_β_1_, and α_6_β_4_ in non-immune cells [reviewed in Ref. (73)]. Nonetheless, there is some evidence for a role of CD151 within immune cells, where the principal β_1_ integrin is α_4_β_1_ which mediates leukocyte adhesion to the ECM protein fibronectin and the endothelial cell adhesion molecule VCAM-1. Early studies of the role of CD151 in the immune system focused on CD151 expressed by neutrophils. Inhibition of CD151 *via* function-blocking antibodies abolished neutrophil migration on the ECM protein fibronectin (13). However, as tetraspanins exist in supramolecular complexes, it is not clear what physiological processes monoclonal antibody (mAb) cross-linking may be mimicking. As such, we have long urged caution in attributing functions to tetraspanins based solely on the use of mAbs (80).

Nonetheless, recent molecular analyses of CD151 function in T cells suggest that CD151 does indeed regulate α_4_ integrin adhesiveness and immune cell migration (14). This paper, by Zelman-Toister and colleagues, eloquently described CD151-integrin interactions in T cells and showed that low-dose CCL2 modulated CD151 expression and cell migration (14). Immunoprecipitation analyses of T cells exposed to CCL2 revealed CCL2-dependent dissociation of CD151/α_4_β_1_ complexes. In addition, ligation of CD151 on the surface of mouse T cells induced actin polymerization through Vav1 phosphorylation and elevated CCL21-induced T cell migration (14). Finally, CD151 ablation on T cells was shown to protect mice from experimental colitis, a result confirmed by interruption of CD151:CD151 associations using an antagonistic peptide to the CD151 LEL (14). The latter reagent resulted in impaired T cell actin remodeling and chemotactic migration *in vitro*. As CD151 has been shown to directly interact with integrin α but not β chains (81), and as α_4_β_7_ is known to be critical for immune cell recruitment to the gut, it is tempting to interpret these data as an effect of CD151 on α_4_β_7_ rather than α_4_β_1_ function. However, a biochemical interaction between any tetraspanin and α_4_β_7_ integrin has not been reported. Nonetheless, these findings illustrate that the CD151 tetraspanin directly affects leukocyte migration and importantly that this role extends to the capacity to influence inflammatory responses.

Finally, the tetraspanin CD82 has also been reported to interact with several integrins including α_4_β_1_ (69, 82). In hemopoietic stem cells (HSC), the CD82/α_4_β_1_ axis has functional relevance. These two molecules colocalize to a polarized membrane domain that has been implicated in mediating HSC adhesion to osteoblasts. Treating HSCs with mAbs against CD82 impairs both their homing to the bone marrow and adhesion to osteoblasts (83). Elegant analyses by super-resolution microscopy in a transfection system using a leukemic progenitor cell line demonstrated that CD82 expression promoted adhesion to fibronectin by promoting both the stability of α_4_β_1_ at the cell surface and the formation of high avidity α_4_β_1_ clusters (84).

Certainly, CD37, CD81, CD82, and to a lesser extent, CD151 are co-expressed in many immune cell types. Why there appears to be functional overlap where all four tetraspanins can promote α_4_β_1_ clustering and adhesiveness and whether there is interplay between the tetraspanins in regulating this integrin is not known. It will be interesting to determine whether these tetraspanins exist within the same microdomain together with α_4_β_1_, or whether distinct tetraspanin/α_4_β_1_ complexes exist.

### β_2_ Integrins

In contrast to the well-described interactions between tetraspanins and the β_1_ integrins, the literature implicating molecular and functional interaction between tetraspanins and β_2_ integrins is less extensive. Nonetheless, CD63, CD82, and CD9 have all been reported to interact with β_2_ integrins (4, 70, 85–87). The functional consequences of the interactions of CD63 and CD82 with β_2_ integrins remain to be defined, although CD82 overexpression has been implicated in LFA-1-dependent homotypic and heterotypic cell–cell adhesion (88). However, the CD9/LFA-1 interaction on monocyte and T cell lines has been confirmed using various techniques including co-precipitation with chemical cross-linking and proximity ligation assays. This association is mediated *via* interaction of the β_2_ subunit of LFA-1 with the LEL of CD9 and is of functional significance, as CD9 negatively regulates LFA-1 adhesive function. However, the mechanism is not fully elucidated, as CD9 did not affect inside-out signaling or display of high affinity integrin, although it was suggested that CD9 modulates LFA-1 clustering (7). Whether this interaction has a functional impact on leukocyte migration *in vivo* remains unknown. Deletion of CD9 was observed to restrict neutrophil and macrophage migration in experimental colitis, an effect consistent with dysregulated LFA-1 function. However, further analyses using bone marrow chimeric mice demonstrated that CD9 expressed on non-hematopoietic cells, rather than leukocytes, was required for disease attenuation (89). Given this observation, the importance of leukocyte-expressed CD9 in immune cell migration remains to be determined.

Perhaps the best evidence for the functional regulation of β_2_ integrins comes from analysis of the CD37^−/−^ mouse. CD37^−/−^ neutrophils display reduced capacity to adhere to β_2_ integrin ligands *in vitro*. *In vivo*, CD37^−/−^ neutrophils also displayed reduced chemokine-induced adhesion in postcapillary venules, as well as dysregulated directional migration in response to chemotactic stimuli. Deletion of CD37 reduced the stability of integrin expression on the surface of activated neutrophils, by promoting an increase in the rate of β_2_ integrin internalization (8). Thus, CD37 constitutively acts to retain β_2_ integrins on the cell surface, a function that would act to stabilize leukocyte adhesion. However, despite these findings of a functional link between CD37 and the β_2_ integrins, super-resolution microscopy analyses failed to reveal significant co-clustering of CD37 and the β_2_ integrin. Furthermore, the absence of CD37 did not affect β_2_ integrin clustering (8). Further experiments will be required to understand the molecular basis of this functional interaction.

## Tetraspanins as Regulators of the Cytoskeleton and DC Migration

How then can a tetraspanin like CD37, which does not colocalize with the β_2_ integrin and does not regulate β_2_ integrin clustering, control integrin adhesiveness, and internalization? A recurring theme in the literature is the concept that the cytoskeletal rearrangements required for cellular polarization, spreading, adhesion, and migration are under the influence of CD37. Indeed, both neutrophils (8) and DCs (63) from CD37^−/−^ mice have been found to be impaired in their capacity to spread and form membrane protrusions on adhesive substrates, processes which are actin-dependent. CD37^−/−^ DCs also displayed impaired adhesion to fibronectin, as well as impairments in migratory function (63). Together, these findings raise the possibility that CD37 functions as a molecular link between integrins and the cytoskeleton, possibly by regulating signaling through the Rho GTPase Rac1 (9). However, the observation that CD37^−/−^ DCs display reduced migration to lymph nodes (63), behavior that can occur in the absence of integrins (43), indicates that CD37 may also regulate integrin-independent migration. The CD81^−/−^ DC phenotype is strikingly similar to that of CD37^−/−^ DCs, in that CD81^−/−^ DCs are also unable to form Rac1-dependent membrane protrusions and show impaired motility and reduced Rac1 activation (11).

However, while migration of CD81^−/−^ DCs on two-dimensional substrates was impeded, their migration in three-dimensional collagen gels was equivalent to that of wild-type DCs (11), indicating that the contribution of CD81 to DC migration is variable, according to the migratory field being encountered. Moreover, these observations are reminiscent of studies showing that DC migration in three-dimensional substrates is unimpeded in the absence of integrins, being dependent instead on actin-mediated cellular contraction and protrusion (43). One possible explanation for this unexpected finding is that in the absence of integrins, actin polymerization and retrograde flow are increased, compensating for reduced capacity to attach to the ECM (90). Interestingly, Quast et al. also observed increased retrograde actin flow in CD81^−/−^ DCs (11). Together, these similar observations seen in the absence of CD81 and integrins provide further evidence of an intimate functional association between these molecular pathways.

By contrast, DCs lacking expression of CD82 are hypermigratory, as shown in both *in vitro* chemotactic assays and *in vivo* lymph node homing assays (9). Like CD37^−/−^ and CD81^−/−^ DCs, CD82^−/−^ DCs lack membrane protrusions, but in contrast, spread to a greater extent than wild-type DCs upon adhesion to fibronectin. Thus, the CD82^−/−^ phenotype is associated with a defect in actin polymerization, likely brought about by dysregulation of signaling through another Rho GTPase, RhoA (9). This integrated relationship between CD82 and the cytoskeleton is reminiscent of previous investigations in T cells. Here, CD82 was found to colocalize with F-actin in lipid rafts (71), and cross-linking CD82 induced dynamic morphological changes such as pseudopodia formation, that are dependent on Rho GTPase activity (72, 91).

The precise molecular interactions by which tetraspanins regulate the cytoskeleton are not understood for CD37 and CD82. On the other hand, in T cells, CD81, *via* its cytoplasmic tail, can interact with Rac1 (92). A further key mechanism bridging membrane proteins to cytoskeletal actin is the ezrin/radixin/moesin (ERM) proteins (93, 94). Tetraspanins CD9 and CD81 have been shown to interact directly with ERM proteins in immune cells (95). In NK cells, cross-linking of CD81 induces phosphorylation of ERM proteins and colocalization of CD81 with phosphorylated ERM at uropods. This is associated with increased cell polarization and migration toward chemoattractants (64). Similarly in B cells and human PBMCs, CD81 cross-linking induces Syk-dependent ezrin phosphorylation and CD81 colocalization with ezrin and polymerized actin (65). Deletion of the C-terminal tail of CD81 resulted in reduced ezrin phosphorylation, providing clear evidence that the association between these molecules impacts on ERM function.

## Influence of Tetraspanins on Endothelial Cell Adhesive Function

As leukocytes are required to undergo extensive interactions with, and eventually transmigrate through, the endothelium during the process of recruitment, adhesive function of endothelial cells is equally as important as that of leukocytes in facilitating an appropriate inflammatory response. In addition to their effects in leukocytes, multiple members of the tetraspanin family have been shown to act in endothelial cells to modulate their capacity to support interactions with leukocytes (Figure 3). For instance, CD9 silencing in human umbilical vein endothelial cells (HUVECs) led to decreased ICAM-1 expression and abrogated leukocyte adhesion and transendothelial migration under flow conditions (58). Subsequent analysis of this phenomenon revealed that both CD9 and CD151 are integral to the formation of membrane structures termed endothelial adhesive platforms (EAPs) in which ICAM-1 and VCAM-1 cluster in the endothelial cell membrane at contact sites with adherent leukocytes (59). This leads to increased avidity for leukocyte integrin ligands and tetraspanin-dependent promotion of leukocyte adhesion and transmigration. The role of CD9 in promoting endothelial cell adhesive function was further examined in a study that used atomic force microscopy to examine the morphology of the endothelial surface at high resolution (60). In response to TNF stimulation, F-actin-containing microvilli decorated with ICAM-1 formed on the endothelium. While these structures developed in the absence of adherent leukocytes, they were thought to serve as precursors for EAPs that form around adherent immune cells (58). CD9 was also incorporated in these structures, and CD9-siRNA studies demonstrated that their formation required CD9. These studies provide further evidence for a role for CD9 in endothelial cells in supporting leukocyte adhesion and recruitment (60).

Endothelial cell-expressed CD81 has also been shown to contribute to leukocyte–endothelial cell interactions. In early atherosclerotic lesions, where monocyte–endothelial interactions are increased, endothelial cells express CD81 at elevated levels (66). *Via* confocal microscopy of TNF-treated endothelial cells, CD81 was found to colocalize with both VCAM-1 and ICAM-1 at contact sites with monocytes. Furthermore, forced expression of CD81 in endothelial cells was sufficient to increase monocyte adhesion to endothelial monolayers, notably without a requirement for stimulation of the endothelium with inflammatory mediators. This increased adhesion was dependent on endothelial ICAM-1 and VCAM-1 but occurred in the absence of upregulation of these adhesion molecules (66). By contrast, overexpression of CD81 increased clustering of ICAM-1 and VCAM-1, with this increased avidity likely to facilitate monocyte adhesion. These findings indicate a role for CD81 in the redistribution of ICAM-1 and VCAM-1 into adhesion-supporting clusters within the endothelial cell membrane (66). This is paralleled by studies in T cells which demonstrate that CD81 influences leukocyte recruitment by promoting integrin avidity (10, 67).

Weibel–Palade bodies in endothelial cells are secretory vesicles that contain von Willebrand factor and P-selectin. These vesicles are released upon endothelial cell activation and aid in promotion of leukocyte rolling in response to acute inflammatory stimuli and hemostasis (96, 97). It has been long established that the tetraspanin CD63 is an additional major component of these structures, although its function was not clear. This was addressed in studies in which CD63 expression was silenced in HUVECs using siRNA (5). CD63-deficient HUVECs showed a reduced capacity to support leukocyte rolling, findings supported by *in vivo* analyses of leukocyte rolling in postcapillary venules of CD63^−/−^ mice. The nature of the association between CD63 and P-selectin was examined using immunogold scanning electron microscopy in HUVECs, revealing that CD63 clustered with P-selectin on the endothelial cell surface. In addition, proximity ligation assays in HEK293 cells showed that surface CD63 and P-selectin colocalized within 20–30 nm of each other (5). Finally, in the absence of CD63, both P-selectin clustering and the level of P-selectin surface expression were reduced relative to non-silenced cells. This indicated that CD63 is a molecular partner of P-selectin and supports its clustering with this being essential for the capacity of P-selectin to mediate rolling.

Metalloproteases (MP) are also tetraspanin-partner proteins. For example, CD9 is a molecular partner and negative regulator of MP ADAM17 (61), substrates of which include ICAM-1 and L-selectin (98). Similarly, the TspanC8 subfamily of tetraspanins (consisting of six members: Tspan5, 10, 14, 15, 17, and 33) have been reported to regulate ADAM10 (99), the targets of which include Notch proteins, amyloid precursor protein associated with Alzheimer’s disease (100), and adhesion molecules. In regards to leukocyte recruitment, TspanC8 members Tspan5 and Tspan17 promote transmigration of T cells by regulating cleavage of VE-cadherin on endothelial cells (12). VE-cadherin is an ADAM10 substrate, and its cleavage is a necessary step toward the completion of T cell transmigration (101). Reyat et al. demonstrated that endothelial ADAM10 function is regulated by Tspan5 and Tspan17, and that silencing of these tetraspanins in HUVECs resulted in inhibition of T lymphocyte transmigration (12). The mechanisms whereby TspanC8 subgroup tetraspanins regulate ADAM10 activity are thought to include effects on intracellular trafficking, promoting ADAM10 exit from the endoplasmic reticulum and enzymatic processing, promoting cleavage of ADAM10 into its mature form (102).

Tetraspanins are also known to be highly expressed on extracellular vesicles (EVs) (103). EVs are membrane-bound subcellular particles released by a wide range of cells, including immune cells such as DCs, macrophages, B cells and endothelial cells (104, 105). Exosomes, EVs in the ~50–100 nm diameter range, are enriched in CD9, CD37, CD53 CD63, CD81, and CD82, with the relative abundance of different tetraspanins varying according to the cell of origin. In many cases, these particles also carry adhesion molecules (103). Furthermore, in some circumstances, EVs have been shown capable of modulating immune cell migration (106–110). However, whether the tetraspanins contained within EVs contribute to this effect on immune cell migration requires further investigation.

## Future Directions

Other tetraspanins may also be worthy of investigation for their actions in leukocyte recruitment. For example, a small case study of individuals deficient in the leukocyte-restricted tetraspanin CD53 revealed that they were affected by recurrent bacterial infections (111, 112). This observation is consistent with this genetic defect resulting in a form of immune deficiency. As effective combat of bacteria by neutrophils is heavily reliant upon their migratory capacity, these observations raise the yet to be investigated possibility of a role for CD53 in these activities.

There is no question that the tetraspanin family of transmembrane molecules is instrumental in ensuring the correct functioning of proteins and processes involved during immune cell migration. Of particular interest is the ability of tetraspanins to functionally associate with the cytoskeleton and influence the remodeling of actin filaments to produce extensions such as lamellipodia and filopodia, structures which are required for leukocyte recruitment and migration. As detailed, some tetraspanins, such as CD82, can elicit inhibitory effects on cell migration, while others, including CD37 and CD151, are able to enhance recruitment events. Though research in this field has documented numerous tetraspanin partners and downstream signaling pathways modulated by these interactions, there is still much to be learnt about the significance of these interactions during immune cell migration. Further investigation into tetraspanin regulation of localization and clustering of integrins and other adhesion molecules in the cell membrane are warranted. In addition, an important distinction that needs to be made is how applicable these mechanisms of regulation are to different immune cell subsets, as tetraspanins have been repeatedly demonstrated to mediate different functions in different cell types. Finally, and most importantly, the influence of these functions on leukocyte recruitment and behavior *in vivo* during inflammatory responses must be examined, to determine if these molecules have potential as therapeutic targets in inflammatory disease.

## Author Contributions

LY wrote the initial draft of the manuscript and was involved in its editing. MH is an expert in leukocyte recruitment and had particular responsibility for the parts of the manuscript dealing with this topic. He had a major role in the editing of the manuscript and made the figures. MW is an expert in tetraspanin immunology and had particular responsibility for the parts of the manuscript dealing with this topic. He also made suggestions to amend the figures and edited the manuscript.

## Conflict of Interest Statement

The authors declare that the research was conducted in the absence of any commercial or financial relationships that could be construed as a potential conflict of interest.
